# Genetic manipulation technologies for precise cellular control: a comparative review of five approaches

**DOI:** 10.3389/fbioe.2026.1818170

**Published:** 2026-04-17

**Authors:** Qin Xiao, Panpan Sun, Jing Liu, Jia Li, Xueqin Xu, Ying Cao, Yanqiong Wu, Changbin Ke

**Affiliations:** 1 Department of Anesthesiology, Institute of Anesthesiology & Pain (IAP), Taihe Hospital, Hubei University of Medicine, Shiyan, Hubei, China; 2 The First Clinical College, Hubei University of Medicine, Shiyan, Hubei, China; 3 Department of Pain, Taihe Hospital, Hubei University of Medicine, Shiyan, Hubei, China

**Keywords:** chemogenetics, genetic manipulation, magnetogenetics, neuromodulation, odorgenetics, optogenetics, sonogenetics

## Abstract

Genetic manipulation technologies have revolutionized our ability to control cellular activities with high precision. Five major approaches—chemogenetics, optogenetics, odorgenetics, magnetogenetics, and sonogenetics—offer distinct advantages for different research and therapeutic scenarios. However, a unified framework for systematic comparison across these technologies has been lacking.This review provides a comprehensive analysis of these five genetic manipulation technologies from three perspectives: molecular mechanisms, quantitative performance metrics, and application scenarios. We first dissect the signaling pathways and key molecular components underlying each technology. We then establish a seven-dimensional evaluation framework encompassing spatiotemporal resolution, tissue penetration, cell-type specificity, reversibility, multiplexing capability, biosafety, and technical accessibility. Using this framework, we systematically score and compare the five technologies, revealing that optogenetics excels in spatiotemporal precision (millisecond/micrometer scale), chemogenetics offers superior clinical translatability, while sonogenetics and magnetogenetics provide advantages for non-invasive deep tissue applications. We further analyze optimal application scenarios for each technology, including neural circuit dissection, chronic disease management, and deep tissue intervention.This comparative analysis provides researchers with an evidence-based guide for technology selection. We propose that future developments should focus on hybrid approaches combining the strengths of multiple technologies, and on addressing current limitations in delivery efficiency and long-term biosafety for clinical translation.

## 1 Introduction

Precise control of cellular activity is fundamental to understanding biological systems and developing therapeutic interventions (Deisseroth, 2011). Over the past 2 decades, genetic manipulation technologies have emerged as powerful tools that combine genetic targeting with external stimuli to achieve cell-type-specific modulation (Armbruster et al., 2007; Boyden et al., 2005). These technologies share a common design principle: genetically encoding an actuator protein that responds to a specific stimulus (light, chemicals, odors, magnetic fields, or ultrasound), thereby converting external signals into defined cellular responses (Stanley et al., 2012; Ibsen et al., 2015).

Five major genetic manipulation technologies have been developed, each utilizing distinct physical or chemical modalities. Chemogenetics, initially conceptualized through RASSL systems and later refined through DREADD technology, employs engineered receptors activated by otherwise inert ligands (Coward et al., 1998). Optogenetics, introduced by Boyden et al., in 2005, uses light-sensitive opsins for millisecond-scale control. More recently, magnetogenetics and sonogenetics have enabled non-invasive deep tissue modulation through magnetic fields and ultrasound, respectively (Tufail et al., 2010). In 2023, odorgenetics was introduced as a novel approach utilizing odorant receptors for chemical-genetic control with potential advantages in receptor diversity and ligand accessibility (Li et al., 2025; Wu et al., 2025).

Despite significant advances in individual technologies, systematic cross-technology comparisons remain scarce. Existing reviews typically focus on single technologies or provide qualitative descriptions without standardized metrics. This gap hinders researchers from making informed decisions when selecting appropriate tools for specific experimental or therapeutic applications. Key questions remain unanswered: How do these technologies compare in terms of spatiotemporal resolution? What are the trade-offs between invasiveness and control precision? Which technology is best suited for a given application scenario?

To address these gaps, this review provides a systematic comparison of five genetic manipulation technologies using a unified analytical framework. We first examine the molecular mechanisms underlying each approach (Section 2). We then introduce a seven-dimensional quantitative evaluation model and present comparative performance data (Section 3). Finally, we analyze scenario-specific technology selection strategies and discuss future directions for the field (Section 4).

This framework aims to serve as a practical guide for researchers and clinicians in technology selection.

## 2 Molecular mechanisms of five genetic manipulation technologies

**FIGURE 1:**
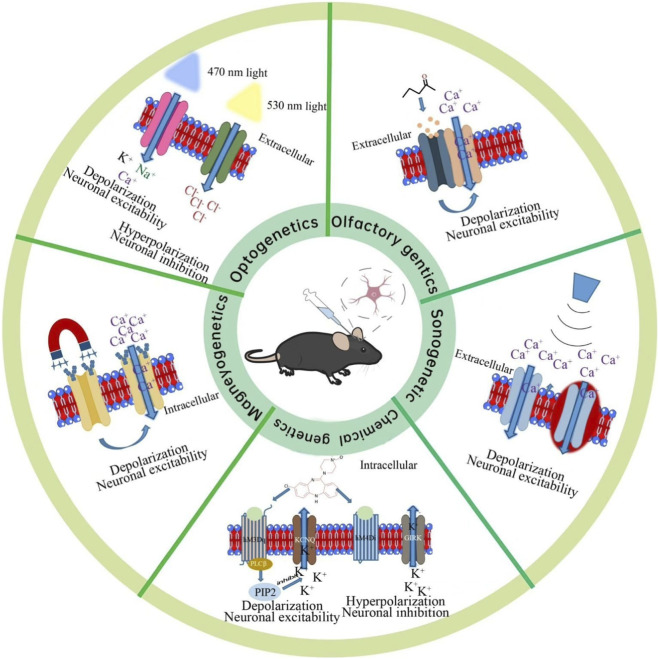
Schematic illustration of core mechanisms for five genetic manipulation technologies.

Although the five genetic manipulation technologies all follow the core logic of “targeted delivery of exogenous responsive elements–specific external signal triggering–activation of intracellular signaling pathways–precise regulation of cellular functions”, they exhibit significant differences in the type of regulatory signals, core functional components, molecular action pathways, and regulatory characteristics (Liu et al., 2025; Zhi et al., 2025; He et al., 2025). These differences directly determine their performance advantages and scenario adaptation tendencies. Below, we analyze the core molecular mechanisms, key technological breakthroughs, and inherent limitations of each technology one by one, laying a foundation for subsequent performance comparison and scenario adaptation analysis (Figure 1).

### 2.1 Chemogenetics

#### 2.1.1 Development and core components

Chemogenetics emerged from efforts to achieve ligand-gated control of cellular activity with high specificity. The technology evolved through several generations: early allele-specific approaches using modified kinases (Bishop et al., 2000), followed by pioneering receptor-based systems such as RASSLs, and culminating in the widely adopted DREADD (Designer Receptors Exclusively Activated by Designer Drugs) platform. The core components consist of engineered G-proteincoupled receptors (GPCRs) that are unresponsive to endogenous ligands but selectively activated by synthetic compounds such as clozapine-N-oxide (CNO) or newer ligands including deschloroclozapine (DCZ) and JHU37160 (Bonaventura et al., 2019; Nagai et al., 2020).

#### 2.1.2 Molecular signaling mechanism

DREADD-based chemogenetics operates through canonical GPCR signaling cascades. The excitatory hM3Dq receptor couples to Gαq proteins, activating phospholipase C (PLC) to hydrolyze phosphatidylinositol 4,5-bisphosphate (PIP2) into inositol trisphosphate (IP3) and diacylglycerol (DAG). IP3 triggers calcium release from the endoplasmic reticulum, while DAG activates protein kinase C (PKC), collectively leading to neuronal depolarization (Alexander et al., 2009). Conversely, the inhibitory hM4Di receptor couples to Gαi proteins, suppressing adenylyl cyclase activity, reducing cAMP levels, and activating G proteincoupled inwardly rectifying potassium (GIRK) channels to hyperpolarize neurons. The KORD (κ-opioid receptor DREADD) system provides an orthogonal inhibitory pathway activated by salvinorin B, enabling multiplexed control when combined with muscarinic DREADDs (Vardy et al., 2015).

#### 2.1.3 Spatiotemporal characteristics

Chemogenetics operates on a timescale of minutes to hours, with onset typically occurring 15–30 min after systemic ligand administration and effects persisting for 6–10 h depending on ligand pharmacokinetics (Smith et al., 2021). Spatial resolution is determined by the genetic targeting strategy (promoter specificity, viral serotype tropism, or intersectional approaches) rather than the stimulation modality, typically achieving regional to cell-type-level precision. A key advantage is the ability to achieve whole-brain or systemic modulation through peripheral ligand administration, enabling noninvasive deep tissue access (Sternson and Roth, 2014).

#### 2.1.4 Current limitations

Several challenges constrain chemogenetic applications: CNO undergoes reverse metabolism to clozapine, which exhibits off-target activity at endogenous receptors; temporal resolution is inherently limited by ligand pharmacokinetics, precluding millisecond-scale control; repeated ligand administration may induce receptor desensitization or immune responses (Urban and Roth, 2015); and achieving precise dose-response relationships requires careful titration due to variable receptor expression levels across animals (Sternson and Roth, 2014).

#### 2.1.5 Challenges and maturity of reproducibility

Chemogenetics boasts excellent reproducibility that has been fully validated across the globe, thus earning well-recognized acceptance in the field of neuroscience. Core variants, including the excitatory subtype (hM3Dq) and inhibitory subtype (hM4Di), have undergone cross-validation in tens of thousands of studies worldwide over decades, with their performance parameters exhibiting remarkable stability (Kang et al., 2023). Moreover, the supporting viral vectors and ligands are available as mature commercial products, and the experimental protocols have been standardized as universal guidelines in the field. Although batch-to-batch variations and metabolic differences of ligands may compromise reproducibility, this limitation has been effectively addressed by next-generation ligands such as DCZ (Kovács et al., 2024). To date, this technology has been well established and widely applied in diverse model organisms, ranging from invertebrates and lower vertebrates to rodents, large animal models, non-human primates, and even humans (Nagoshi et al., 2024).

### 2.2 Optogenetics

#### 2.2.1 Development and core components

Optogenetics was established in 2005 when Boyden et al.demonstrated that the algal protein Channelrhodopsin-2 (ChR2) could depolarize mammalian neurons upon blue light illumination with millisecond precision. This breakthrough was followed by the discovery and engineering of diverse opsin variants: inhibitory halorhodopsins (NpHR) and archaerhodopsins (Arch) for neuronal silencing (Zhang et al., 2007; Chow et al., 2010), redshifted variants (Chrimson, ReaChR) for deeper tissue penetration (Klapoetke et al., 2014), and step-function opsins (SFOs) for bistable control (Berndt et al., 2009). The core components include light-sensitive microbial opsins, optical delivery hardware (lasers, LEDs, fiber optics), and genetic targeting elements (Fenno et al., 2011).

#### 2.2.2 Molecular signaling mechanism

Opto genetic manipulation technologies function through direct light-gated ion flux, bypassing second messenger cascades. ChR2 and its variants are light-gated cation channels that conduct Na^+^, K^+^, Ca^2+^, and H^+^ upon photon absorption by the retinal chromophore, causing rapid membrane depolarization (Nagel et al., 2003). The photocycle involves retinal isomerization (all-trans to 13-cis), channel opening within microseconds, and thermal relaxation back to the closed state within milliseconds. Inhibitory opsins operate through distinct mechanisms: halorhodopsins are light-driven Cl^−^ pumps that hyperpolarize neurons, while archaerhodopsins are outward H^+^ pumps. This direct ion channel mechanism underlies the exceptional temporal precision of optogenetics.

#### 2.2.3 Spatiotemporal characteristics

Optogenetics achieves unparalleled spatiotemporal resolution among genetic manipulation technologies. Temporal precision reaches the millisecond scale, with ChR2 channel opening occurring within 1–2 ms of light onset and closing within 10–20 ms after light offset. Advanced variants such as Chronos achieve sub-millisecond kinetics. Spatial resolution can reach single-cell or even subcellular levels when combined with two-photon illumination and soma-targeted opsin variants (Packer et al., 2012; Shemesh et al., 2017). However, tissue penetration is fundamentally limited by light scattering and absorption, restricting effective stimulation depth to approximately 1–2 mm for blue light, though red-shifted opsins extend this range modestly (Zhang et al., 2008).

#### 2.2.4 Current limitations

Despite its precision, optogenetics faces several constraints: (1) invasive hardware requirements, including chronic fiber implantation for deep brain applications, which causes tissue damage and limits clinical translation; (2) light-induced heating can cause non-specific neuronal activation or tissue damage at high intensities; (3) continuous high-frequency stimulation may cause opsin desensitization or phototoxicity; and (4) the requirement for exogenous retinal supplementation in some non-neuronal cell types limits application scope (LaLumiere, 2011).

#### 2.2.5 Challenges and maturity of reproducibility

Optogenetics exhibits a high degree of reproducibility owing to its clear mechanism, predictable effects, standardized components and supporting system chains, as well as fully quantifiable and controllable manipulation data. To date, extensive reproducible data have been obtained across a broad spectrum of research contexts, ranging from *Drosophila* and rodents to even human clinical trials, making it one of the most widely applied neuromodulation technologies currently available (Diester et al., 2011). Across diverse model organisms and multiple biological scales and biological levels, optogenetics serves as the gold-standard technology among the five techniques, having undergone the most comprehensive validation and boasting the broadest application scope—its mature applications span from *in vitro* single-cell experiments to non-human primate studies and human clinical trials (Sahel et al., 2021).

### 2.3 Odorgenetics

#### 2.3.1 Development and core components

Odorgenetics is a recently developed genetic manipulation technology, with a notable implementation reported by Wu, Ke et al., in 2025 utilizing the *Drosophila* odorant receptor system (DORs). Unlike earlier attempts using mammalian G protein-coupled receptors, this system exploits the unique properties of insect olfactory receptors. The core components consist of a heteromeric complex formed by a specific ligand-binding subunit (e.g., OR35a) and an obligate co-receptor (Orco). These two components must be co-expressed to form a functional unit. The system was designed to provide a minimally invasive, “fast on/off” tool for physiological manipulation, distinguishing it from the slower kinetics of conventional chemogenetics (Wu et al., 2025).

#### 2.3.2 Molecular signaling mechanism

The fundamental mechanism of the DORs-based odorgenetics differs significantly from mammalian olfactory signaling. While mammalian odorant receptors are GPCRs that rely on second messenger cascades (cAMP), *Drosophila* odorant receptors function as ligand-gated ion channels (ionotropic receptors) (Sato et al., 2008). Upon binding of specific odorant ligands (e.g., menthol or other volatile compounds) to the OR35a subunit, the OR35aOrco heteromeric complex undergoes a conformational change that directly opens a non-selective cation pore (Butterwick et al., 2018). This allows the rapid influx of Ca^2+^ and Na^+^ ions, leading to immediate membrane depolarization and calcium signaling. This direct gating mechanism bypasses the latency associated with G protein coupling, enabling the system to achieve rapid activation and deactivation kinetics. Additionally, the functional expression and regulation of this insect-derived complex in mammalian cells require precise genetic engineering. To ensure the optimal 1:1 stoichiometric co-expression of the OR35a and Orco subunits, multicistronic vectors utilizing self-cleaving 2A peptides (e.g., P2A or T2A) or internal ribosome entry sites (IRES) are typically employed. Promoter selection is also critical for regulatory efficiency; while ubiquitous promoters (e.g., CAG or CMV) yield high overall expression, cell-type-specific promoters (e.g., hSyn for neurons) are essential for targeted modulation. A major regulatory challenge in mammalian systems remains the membrane trafficking and localization efficiency of these heterologous proteins, as the absence of native insect chaperones may limit the density of functional receptors on the mammalian cell surface (Benton et al., 2006; Martínez-Salas, 1999; Szymczak et al., 2004).

#### 2.3.3 Spatiotemporal characteristics

A defining feature of the DORs system is its “fast on/off” kinetics, operating on a timescale of seconds. Wu et al. demonstrated that calcium transients can be triggered within seconds of odorant delivery and return to baseline rapidly upon odorant removal, offering a temporal resolution superior to GPCR-based chemogenetics (which typically operates on minutes-to-hours scales).Spatially, the resolution depends on the odorant delivery method. Systemic inhalation allows for broad, non-invasive modulation of targeted tissues (e.g., brain or heart), while localized delivery can achieve regional specificity. The high sensitivity of the system allows effective activation even with low concentrations of volatile odorants (Wu et al., 2025).

#### 2.3.4 Current limitations

Despite its advantages in speed and invasiveness, the technology faces specific challenges: (1) Heterologous expression: Functional expression of insect ion channels in mammalian cells requires the precise co-expression of both OR and Orco subunits, which may vary across cell types (Sato et al., 2008) (2) Delivery precision: While inhalation is non-invasive, precise quantification of the effective concentration at the target tissue depth can be difficult due to odorant volatility and diffusion; (3) Immunogenicity: As *Drosophila* proteins, OR35a and Orco are foreign to the mammalian immune system, potentially limiting long-term applications due to immune rejection; and (4) Ligand selectivity: While designed for specific odorants, potential off-target activation of endogenous mammalian sensory receptors by the volatile ligands requires careful control.

#### 2.3.5 Challenges and maturity of reproducibility

Odorgenetics is a newly developed gene modulation technology in recent years with a clear and uncontroversial mechanism. When odorants are delivered via nasal routes, delivery efficiency and effective concentration at target neurons can be sensitive to dosing/airflow/animal state, which raises the bar for procedural control and cross-experiment consistency. While the core biological effects of the technology itself are reproducible, the threshold for controlling variables in experimental procedures is high (Chin et al., 2018; Stensmyr et al., 2012). To date, odorgenetics has only been applied in specific research scenarios, and universal standardized experimental protocols have not yet been established across the industry (Tumkaya et al., 2022). Therefore, odorgenetics faces a moderate level of reproducibility challenges compared with optogenetics and chemogenetics. This technology has been applied in *Drosophila* and mice (Wu et al., 2025).

### 2.4 Magnetogenetics

#### 2.4.1 Development and core components

Magnetogenetics emerged from the pursuit of non-invasive deep tissue neuromodulation using magnetic fields. The technology leverages magnetically sensitive proteins or protein-nanoparticle complexes to convert magnetic stimuli into cellular responses. Two main strategies have been developed: (1) ferritin-based systems that fuse the iron-storage protein ferritin to temperature- or mechanosensitive channels (Wheeler et al., 2016; Stanley et al., 2015); and (2) magnetite-based approaches using biogenic or synthetic magnetic nanoparticles (Chen et al., 2015). Key molecular components include ferritin (heavy and light chains), thermosensitive TRP channels (TRPV1, TRPV4), mechanosensitive channels (Piezo1), and in some implementations, exogenous magnetic nanoparticles to enhance magnetic responsiveness (Lee et al., 2021).

#### 2.4.2 Molecular signaling mechanism

Magnetogenetic systems operate through magnetothermal or magnetomechanical transduction mechanisms. In magnetothermal approaches, alternating magnetic fields (AMFs) induce heating of ferritin-bound iron oxide nanoparticles or co-administered magnetic nanoparticles, locally activating temperature-sensitive TRPV1 channels (activation threshold ∼42 °C). Channel opening permits Ca^2+^ influx, triggering downstream signaling cascades. In magnetomechanical approaches, magnetic torque or force generated by magnetic particles in static or low-frequency fields is proposed to directly gate mechanosensitive channels. However, the biophysical plausibility of endogenous ferritin generating sufficient force for channel gating remains debated, as theoretical calculations suggest the magnetic moment may be insufficient (Meister, 2016). Recent studies have employed enhanced constructs with increased iron loading or synthetic magnetosomes to improve magnetic sensitivity (Farhadi et al., 2021).

#### 2.4.3 Spatiotemporal characteristics

Magnetogenetics achieves temporal resolution on the scale of seconds to minutes, limited by the kinetics of heat transfer (magnetothermal) or mechanical force transmission (magnetomechanical) and subsequent channel gating. Spatial resolution is typically in the millimeter range, determined by the magnetic field gradient and thermal diffusion properties (Christiansen et al., 2019). A major advantage is the excellent tissue penetration of magnetic fields, enabling non-invasive stimulation of deep brain structures or peripheral organs without surgical implantation. Low-frequency magnetic fields (<1 kHz) penetrate biological tissue with negligible attenuation, theoretically allowing whole-body access.

#### 2.4.4 Current limitations

Magnetogenetics faces significant challenges that have generated controversy in the field: (1) the fundamental question of whether endogenous ferritin generates sufficient magnetic force to activate ion channels under physiological conditions remains unresolved, with some replication studies reporting negative results; (2) magnetothermal approaches require high-intensity AMFs that may cause nonspecific tissue heating; (3) the requirement for specialized magnetic field generators limits accessibility and standardization; (4) temporal resolution is inferior to optogenetics by several orders of magnitude; and (5) the mechanism of action remains incompletely characterized, hindering rational optimization. Despite these concerns, several groups have reported reproducible *in vivo* neuromodulation, suggesting that optimized implementations may overcome current limitations (Christiansen et al., 2019; Xu et al., 2020).

#### 2.4.5 Challenges and maturity of reproducibility

Magnetogenetics remains one of the most controversial neuromodulation approaches in terms of reproducibility, largely because its proposed mechanisms of action have not reached field-wide consensus. Since the initial reports, multiple independent methodological and replication-oriented studies—including those published in high-impact journals—have raised substantial concerns about mainstream protein-based magnetogenetic strategies, reporting little to no reliable magnetic-field–evoked activity under commonly used experimental conditions and highlighting key technical confounds. In parallel, physics-based analyses have argued that several proposed mechanisms face stringent biophysical constraints under practical magnetic field strengths, further contributing to the lack of universal acceptance (Qin et al., 2016). As a result, magnetogenetics is currently best viewed as being at an early proof-of-concept stage, where rigorous mechanism clarification and standardized validation criteria are still needed before stable, routine application can be expected. To date, demonstrations have been reported across several model systems—including *C. elegans*, zebrafish, mice, and non-human primates—but broad, cross-laboratory validation and mature, standardized protocols remain limited compared with optogenetics and chemogenetics (Meister, 2016; Pang et al., 2017).

### 2.5 Sonogenetics

#### 2.5.1 Development and core components

Sonogenetics was developed to harness ultrasound as a noninvasive stimulus for genetically targeted cellular control. The technology was pioneered by Ibsen et al., who demonstrated that the mechanosensitive channel TRP-4 could confer ultrasound sensitivity to *C. elegans* neurons. Subsequent work has expanded the toolkit to include mammalian mechanosensitive channels and engineered variants. Core components include mechanosensitive ion channels (MscL, MscS, Piezo1, TRPV1, TRPA1), ultrasound transducers capable of delivering focused acoustic energy, and in some implementations, gas-filled microbubbles or nanoparticles to enhance mechanical coupling. The technology exploits the ability of ultrasound to generate mechanical forces (radiation force, acoustic streaming, cavitation) that can gate mechanosensitive channels (Yoon et al., 2026; Blackmore et al., 2019).

#### 2.5.2 Molecular signaling mechanism

Sonogenetic transduction occurs through mechanosensitive channel activation by ultrasound-induced mechanical perturbations. Crucially, the functional mechanisms differ significantly depending on specific ultrasonic parameters (frequency, intensity, and pulse width). High-frequency, low-intensity focused ultrasound primarily generates acoustic radiation force and acoustic streaming. These forces induce subtle lateral tension and curvature changes in the lipid bilayer, which directly gate tension-sensitive channels like Piezo1 through its propeller-like blade domains. Conversely, low-frequency, high-intensity ultrasound—often applied with gas vesicles or microbubbles—triggers the cavitation effect. Cavitation generates intense, localized mechanical stress and micro-jets capable of activating channels with higher mechanical thresholds (e.g., MscL). Additionally, the biophysical characteristics of the target channels dictate their regulatory responses. While Piezo1 responds strictly to mechanical membrane deformation, channels like TRPV1 exhibit multi-modal responsiveness; they can be activated not only by ultrasound-induced mechanical stretch but also by localized thermal effects (mild tissue heating) generated during acoustic wave absorption (Ge et al., 2015; Ye et al., 2018). The resulting ion influx (primarily Ca^2+^ and Na^+^) depolarizes the cell membrane and triggers downstream signaling. Gas vesicles or microbubbles can amplify mechanical effects through cavitation, enhancing channel activation at lower ultrasound intensities.

#### 2.5.3 Spatiotemporal characteristics

Sonogenetics achieves temporal resolution in the range of tens to hundreds of milliseconds, intermediate between optogenetics and chemogenetics. The kinetics are determined by ultrasound pulse parameters and channel gating dynamics. Spatial resolution depends on ultrasound focusing capability, typically achieving millimeter-scale precision with conventional transducers, though advanced phased arrays and high-frequency transducers can improve this to sub-millimeter levels. A major advantage is the exceptional tissue penetration of ultrasound, which can reach depths of 10–15 cm in soft tissue with focused delivery, enabling non-invasive access to deep brain structures, peripheral ganglia, and internal organs. This penetration depth substantially exceeds that of lightbased approaches (Rabut et al., 2020; K et al., 2018; Darmani et al., 2022).

#### 2.5.4 Current limitations

Several challenges constrain sonogenetic applications: (1) the sensitivity of current mechanosensitive channels to ultrasound is relatively low, requiring high acoustic intensities that approach safety limits for clinical use (Pasquinelli et al., 2019); (2) ultrasound focusing through the skull is complicated by bone-induced aberrations, requiring patient-specific corrections or limiting applications to post-craniotomy settings (Kyriakou et al., 2014); (3) potential offtarget effects on endogenous mechanosensitive channels expressed throughout the body; (4) gas vesicle or microbubble-enhanced approaches face challenges in stability, biodistribution, and regulatory approval; and (5) the biophysical mechanisms linking ultrasound parameters to channel gating remain incompletely characterized, hindering systematic optimization. Ongoing research focuses on engineering ultrasound-hypersensitive channel variants and developing improved focusing technologies.

#### 2.5.5 Challenges and maturity of reproducibility

The mechanism of sonogenetics remains the subject of widespread controversy, and its reproducibility is limited to highly defined experimental conditions, resulting in low overall reproducibility as universal standardized protocols have not yet been established. This technology has been extensively validated in lower animals and rodents, but only minimally verified in higher animals. As an emerging technique with an unformed standardization system, sonogenetics exhibits a higher overall maturity level than magnetogenetics yet is far less mature than optogenetics/chemogenetics.

### 2.6 Common molecular characteristics and shared technical bottlenecks

Despite utilizing distinct external stimuli (light, chemicals, odors, magnetic fields, or ultrasound), these five genetic manipulation technologies share fundamental molecular characteristics. First, they all strictly rely on the heterologous expression of engineered or non-native actuator proteins (e.g., algal opsins, insect odorant receptors, or bacterial mechanosensitive channels) within mammalian host cells. Second, their downstream signal transduction pathways universally converge on the modulation of ion flux (e.g., Ca^2+^, Na^+^, Cl^−^) or the generation of second messengers (e.g., cAMP, IP3) to ultimately alter membrane potential and cellular activity (Deisseroth, 2011; Benton et al., 2006; Roth, 2016).

Consequently, these shared mechanisms give rise to common technical bottlenecks across all five fields. A primary shared limitation is the immunogenicity of heterologous proteins; the long-term expression of non-human proteins inevitably poses the risk of triggering host immune rejection or neuroinflammation, which severely hinders clinical translation. Additionally, all these technologies are fundamentally constrained by the specificity and efficiency of targeted *in vivo* delivery. Current viral vectors (such as AAVs or lentiviruses) exhibit inherent limitations regarding packaging capacity, tissue tropism, and off-target expression in non-target organs (e.g., liver accumulation) (Mingozzi and High, 2013; Kotterman and Schaffer, 2014). Therefore, achieving absolute cell-type specificity without shared off-target delivery bottlenecks remains a universal challenge that must be addressed in future developments.

This circular diagram centers on the mouse model to visualize the signal-triggered regulatory processes of five genetic manipulation technologies, with each sector corresponding to one technology and its key cellular responses.

1. Optogenetics: Triggered by 470 nm (blue) or 530 nm (yellow) light, membrane-localized light-sensitive proteins (e.g., ChR2, NpHR) mediate cation influx (depolarization, neuronal excitation) or chloride influx (hyperpolarization, neuronal inhibition), respectively.
2. Olfactory genetics: Binding of the exogenous odorant two-pentanone to the OR35a/Orco heterodimeric complex opens the ion channel, inducing calcium influx and cellular depolarization (neuronal excitation).
3. Sonogenetics: Ultrasonic stimulation elicits minor membrane deformation, activating mechanosensitive ion channels to trigger calcium influx and cellular depolarization (neuronal excitation).
4. Chemical genetics: The synthetic ligand CNO binds to engineered hM3Dq/hM4Di receptors, which respectively drive Gqα/PLCβdependent depolarization (neuronal excitation) or Giα/Gβγ-dependent hyperpolarization (neuronal inhibition).
5. Magnetogenetics: External magnetic field stimulation acts on magnetic nanoparticles (MNPs) to activate ion channels, leading to calcium influx and cellular depolarization (neuronal excitation).

This figure intuitively contrasts the signal triggers, core functional components, and cellular response patterns of the five technologies, highlighting their mechanistic divergence in precise genetic manipulation.

## 3 Performance comparison of the five genetic manipulation technologies

To objectively evaluate the comprehensive performance of the five genetic manipulation technologies, we selected seven core indicators—spatiotemporal resolution, penetration depth, reversibility efficiency, invasiveness degree, regulatory duration, equipment dependence, and biological safety—in light of the requirements of basic research and clinical translation, and drawing on the latest research findings (2024–2025) (Zhu et al., 2024). A standardized evaluation system (with a full score of 10 points, where higher scores correspond to superior performance) was established to quantitatively compare the advantages and limitations of each technology, thereby providing a quantitative basis for technology selection.

### 3.1 Definition and scoring criteria of core performance indicators

#### 3.1.1 Definition and scoring methodology of core performance indicators

To ensure objectivity, the scoring framework in Table 1 employs a mixed-methods approach combining literature-derived quantitative metrics and qualitative expert consensus. Objective parameters, such as spatiotemporal resolution and penetration depth, are scored strictly based on quantitative physical and pharmacokinetic data reported in recent literature (Hsu and Sandford, 2007; Tsukada et al., 2021). Conversely, parameters like biosafety and clinical application suitability are evaluated qualitatively based on current technological maturity and reported adverse events. For instance, biosafety risk levels are justified as follows: “Low risk” implies high biocompatibility with extensive validation; “Medium risk” indicates potential side effects (e.g., off-target ligand binding or thermal damage) that require careful parameter control; and “High risk” reflects a current lack of long-term toxicological data or significant mechanistic controversies. Where variability or uncertainty exists across studies, scores reflect the most consistently reported average performance (Guyatt et al., 2008).

**TABLE 1:** presents the core performance indicators and unified scoring criteria for the five genetic manipulation technologies, encompassing seven critical performance dimensions (including spatiotemporal resolution, penetration depth, and biosafety). These standardized criteria enable systematic quantitative assessment of the technical performance profiles of each approach, laying a foundational framework for subsequent comparative evaluation and targeted scenario-specific adaptation.

Indicator	Full score	Scoring criteria
Spatiotem Poral Resolution	7 points (4 for temporal +3 for spatial)	Temporal: Millisecond (4) > Second (3) > Minute (2) > Hour (1); Spatial: Single cell (3) > Tissue block (2) > Organ (1) Note: Higher scores = superior high-precision monitoring (temporal: min data acquisition interval; spatial: min identifiable target size)
Penetration Depth	5 points	<0.2 cm (1); 0.2–2 cm (2); 2–5 cm (3); >5 cm (4); Whole-body (5) Note: Score based on physical accessible range (imaging/implantation); whole-body coverage = highest score (risk mitigation for implants)
Reversibility Efficiency	5 points	0%–100% reversibility = 0–5 points (1 point per 20%) Note: Scored by reversibility degree of technical operations (user-defined rules if no direct data)
Invasiveness Level	3 points	Non-invasive (0); Minimally invasive (1); Implantation (2); Invasive (3) Note: Score by physical interference degree (non-invasive = lowest; surgical implantation = highest; medical device classification as reference)
Modulation Intensity	±3 points	Strong bidirectional (±3); Weak bidirectional (±2); Unidirectional (+2/−2) Note: Score by bidirectional/unidirectional effect range (user-defined rules if no direct data)
Biosafety	5 points	Low risk (5); Medium risk (3); High risk (1) Note: Score by health risks (high biocompatibility = highest; teratogenicity/infection = lowest; biomaterial toxicity/immune response as reference)
Equipment Dependence	4 points	No equipment (0); Portable (1); Laboratory-based (2); Large-scale (4) Note: Score by hardware reliance (large-scale instruments = highest; implantable device equipment hierarchy as reference)

### 3.2 Performance comparison and core conclusions of the five genetic manipulation technologies

#### 3.2.1 Odorgenetics

Odorgenetics employs two-pentanone as the specific ligand. Following inhalational administration, the concentrations of 2pentanone in the blood and cerebrospinal fluid decrease rapidly after drug withdrawal. A study by Wu’s team in Protein Cell confirmed that this ligand can be rapidly eliminated via exhalation, endowing the technology with rapid reversibility for transient regulation. Xiang’s team further verified the safety and reversibility characteristics of this technology in Medical Gas Research.

The tissue penetration depth of odorgenetics is limited to the millimeter scale (<0.2 cm), acting primarily on specific brain regions such as the cerebrospinal fluid and central amygdala. Its core receptors (e.g., the OR35a/Orco complex) require targeted delivery via AAV vector injection, making the gene delivery process a minimally invasive procedure, while the activation approach adopts non-invasive inhalational administration. Currently, odorgenetics only has activated tools developed, with no mature inhibitory regulatory strategies available, thus being categorized as a unidirectional regulatory system. From a biosafety perspective, while immunogenicity of the foreign *Drosophila* proteins (OR35a/Orco) and potential ligand off-target effects on endogenous mammalian receptors are primary concerns, the pharmacokinetic profiles of the volatile ligands must also be scrutinized. The *in vivo* metabolic pathways of volatile ligands like two-pentanone primarily involve hepatic processing via cytochrome P450 enzymes, followed by rapid renal excretion or exhalation. However, comprehensive toxicological data regarding long-term inhalation exposure is currently lacking. Prolonged exposure to such volatile organic compounds (VOCs) may induce respiratory mucosal irritation, localized inflammatory responses, or olfactory receptor desensitization. Because systematic long-term safety and inhalation toxicity assessments have not yet been fully established, odorgenetics is currently evaluated as a high-risk technology (Trimmer et al., 2019; Omura et al., 2022).

#### 3.2.2 Chemogenetics

Chemogenetics relies on ligands such as clozapine-N-oxide (CNO) as its core effectors. These ligands can cross the blood-brain barrier (BBB), with a diffusion range spanning from several millimeters to centimeters. However, their delivery efficiency is subject to dual constraints imposed by BBB permeability and *in vivo* metabolic processes. A study by Gomez et al. in Science demonstrated that this technology requires intravenous or intraperitoneal injection to achieve targeted and efficient delivery, whereas oral administration yields limited efficacy due to poor BBB penetration (Gomez et al., 2017). CNO exerts its regulatory effects following metabolic conversion to clozapine *in vivo*; it has a relatively long time to peak efficacy (two to three h), and the reversibility of its regulation is dependent on the rate of drug clearance. Moreover, clozapine may induce adverse reactions such as agranulocytosis (Campbell and Marchant, 2018; Abanmy et al., 2014).

In terms of regulatory capacity, chemogenetics enables weak bidirectional modulation through the individual or coexpression of engineered receptors including hM3Dq and hM4Di. From a biosafety perspective, the core bottleneck lies in the propensity for off-target effects and retrograde metabolism upon long-term systemic ligand administration. Additionally, systematic long-term toxicological data and reproductive safety assessments remain lacking. Collectively, these factors categorize chemogenetics as a moderate-risk technology (Manvich et al., 2018; Goossens et al., 2021).

#### 3.2.3 Optogenetics

Optogenetics features both millisecond-scale precise manipulation and single-cell targeting capability, with its penetration depth varying considerably with light wavelength. Specifically, visible light (e.g., blue light) achieves a penetration depth of approximately 200–800 μm (<0.2 cm), whereas lightsensitive proteins engineered for long-wavelength light (e.g., ChRmine) can extend the penetration depth to 7 mm (0.7 cm) (Chen et al., 2021a).

Conventional delivery strategies for this technology necessitate the implantation of optical fibers or LED devices to transmit light signals, classifying it as an implantation-grade invasive procedure. A study by Deisseroth’s group in Nature Biotechnology indicated that long-term implantation of such devices may trigger local inflammatory responses. Although non-invasive delivery protocols have been preliminarily explored, implantable strategies still dominate the current applications (Owen et al., 2019).

In terms of regulatory capacity, optogenetics enables rapid, reversible, and robust bidirectional modulation by integrating red-light-driven activating channels and blue-light-driven inhibitory pumps. From the perspective of safety risks, the core hazards stem from invasive operations and light-induced thermal effects (the latter of which has established quantitative control criteria) (Lindner et al., 2022). However, the lack of comprehensive data regarding the potential toxicity of exogenous proteins and their long-term immune responses constitutes a key safety concern. Collectively, optogenetics is evaluated as a moderate-risk technology (White et al., 2020; Harris and Gilbert, 2022).

#### 3.2.4 Magnetogenetics

Magnetogenetics achieves centimeter-scale magnetic field penetration (≤2 cm), enabling whole-brain coverage that encompasses key brain regions such as the medial preoptic area and hypothalamus. A study by the team from the Institute for Basic Science (South Korea, 2024) in Nature Nanotechnology confirmed the favorable reversibility of the Nano-MIND technology: the functional regulatory effect recovers within minutes after magnetic field cessation. Although magnetic nanoparticles may persist *in vivo*, their associated impacts attenuate rapidly (Choi et al., 2024).

The core delivery and regulatory strategy of this technology involve intravenous injection of superparamagnetic Fe_3_O_4_ nanoparticles, which enables targeted modulation upon exposure to external magnetic fields without the need for implanting invasive devices. However, the biodegradability of these nanoparticles remains to be further verified. Regarding regulatory capacity, some studies have reported that “bidirectional regulation” of motor circuits can be achieved, but the intensity and stability of the regulatory effect are relatively weak, with a slow response rate, classifying it as a weak bidirectional regulatory system overall (Long et al., 2015).

Currently, magnetogenetics is plagued by significant fundamental controversies. Additionally, systematic biosafety assessment data are lacking, and potential risks such as thermal effects and longterm magnetic field exposure have not been fully quantified. Collectively, magnetogenetics is evaluated as a high-risk technology.

#### 3.2.5 Sonogenetics

In sonogenetics, following the cessation of ultrasonic stimulation, partial persistence of regulatory effects may occur via Reactive Oxygen Species (ROS)-mediated mechanisms. Coupled with prolonged metabolic clearance, this results in limited reversibility—a phenomenon validated by Ye’s group in Cell Reports Medicine (Gao et al., 2024).

The technical implementation of sonogenetics requires the injection of Adeno-Associated Virus (AAV) vectors to express ultrasound-sensitive channels (e.g., TRPV1 mutants) in target cells; while activation relies on non-invasive external ultrasonic stimulation, the viral vector injection is categorized as a minimally invasive procedure. Although preliminary evidence has demonstrated the potential of bidirectional modulation for this technology, there are currently no definitive reports of controllable excitation and inhibition being achieved with a single ultrasound-sensitive channel under standard parameters and physiological conditions. The robustness and precision of its bidirectional modulation remain to be further validated, thus classifying it as a weak bidirectional regulatory system overall (Yitzhak-David and Rotenberg, 2023; Xian et al., 2023; W et al., 2024).

From a safety perspective, the core risk of sonogenetics stems from tissue damage induced by the ultrasonic cavitation effect. Currently, systematic data on its safe parameter window and the long-term impacts of application remain scarce, leading to its comprehensive evaluation as a moderate-risk technology.

### 3.3 Comprehensive performance summary

**FIGURE 2:**
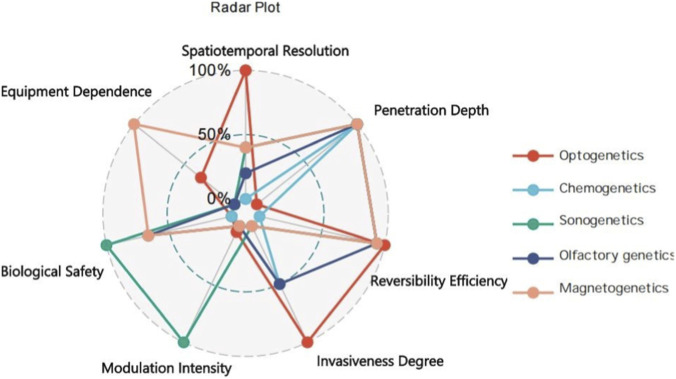
Radar plot comparing the performance profiles of five genetic manipulation technologies.

To illustrate the quantitative discrepancies and overall performance profiles of the five genetic manipulation technologies across seven core evaluation indicators—spatiotemporal resolution, penetration depth, reversibility efficiency, invasiveness degree, modulation intensity, biological safety, and equipment dependence—the systematic scoring-based analysis results outlined earlier are translated into a radar plot (Figure 2). This visualization clearly delineates the advantageous dimensions and performance limitations of each technology, thereby providing an intuitive comparative reference for subsequent technology selection, scenario adaptation, and cross-technology integration.

This radar plot depicts the performance profiles of the five genetic manipulation technologies across seven core indicators. Specifically:

Odorgenetics achieves the highest overall score (6.9) by virtue of its high non-invasiveness (trauma-free via inhalational administration), low equipment dependence (requiring only simple odor generators), and favorable biosafety (rapid metabolism of odorant molecules). It is well-suited for scenarios involving freely moving animals and non-invasive interventions (e.g., preliminary management of respiratory diseases). Chemogenetics excels in regulatory duration (sustained efficacy for several days per administration) and operational convenience (syringe-based administration without sophisticated equipment), with an overall score of 6.7. It is particularly appropriate for investigating long-term physiological processes (e.g., establishment of chronic metabolic disease models).

Optogenetics, despite a relatively low overall score (5.4), is irreplaceable in scenarios demanding ultra-high precision (e.g., neural circuit dissection), as it possesses unmatched spatiotemporal resolution (scoring a full 10 points) and thus serves as the first choice for single-cell precise modulation.

Sonogenetics (6.3) and magnetogenetics (6.0) hold an unrivaled advantage in deep tissue penetration (both scoring 10 points), making them core candidate technologies for clinical deep-organ modulation (e.g., deep brain regions, tumor tissues).

## 4 Scenario adaptation analysis of the five genetic manipulation technologies

Based on the quantitative comparison of the seven core indicators and the comprehensive performance profiles visualized by radar plots, the advantageous dimensions and limiting characteristics of each technology have been clearly delineated—these inherent performance divergences essentially dictate their inclinations for scenario adaptation (Li et al., 2021).

In the subsequent sections, we adhere closely to the intercorrelative logic of mechanism-characteristics scenarios and systematically delineate the specific application scenarios of the five genetic manipulation technologies from two core dimensions: basic research (e.g., neural circuit dissection, experiments on freely moving animals) and translational applications (e.g., treatment of neuropsychiatric disorders, deep organ modulation, regenerative medicine) (Kim et al., 2017). This analysis clarifies the technology selection principles and practical implications for distinct scenarios, thereby providing targeted guidance for basic research design and clinical translation exploration.

### 4.1 Scoring criteria and summary analysis for application scenario adaptation

Based on the core requirements of application scenarios and the three-level scoring logic specified in Table 2, this study further performed quantitative scoring on the adaptability of odorgenetics, chemogenetics, optogenetics, magnetogenetics, and sonogenetics across seven typical scenarios, ultimately yielding a scenario adaptability scoring table for each technology (Table 3). This table intuitively demonstrates the specific scoring discrepancies of different technologies in scenarios including basic research on neural circuits, deep brain region modulation, and local tumor therapy, clearly delineating the scenario adaptation advantages and limitations of each technology. It thereby furnishes researchers with a direct and quantitative reference for rapidly screening optimal technical solutions tailored to their practical research needs.

**TABLE 2:** Comprehensive Scoring Breakdown for the Five Genetic Manipulation Technologies, Scores are based on the criteria defined in Table 1. Max score for each metric is indicated in parentheses. (Note: The normalized scores in the radar plot (Figure 2) are calculated based on weighted averages of these raw metrics to fit a 10-point scale for visual representation.).

Technology type	Spatiotemporal resolution	Penetration depth	Reversibility efficiency	Invasiveness level	Modulation intensity	Biosafety	Equipment dependence	Reference
Odorgenetics	3	1	5	1	+2	1	2	Wu et al. (2025); De March et al. (2023); Noe et al. (2017)
Chemogenetics	2	2	2	1	±2	3	0	Gao et al. (2026); Szablowski et al. (2018); Perez et al. (2022); Corigliano et al. (2025); Yu et al. (2025); Wang et al. (2025)
Optogenetics	7	2	5	2	±3	3	2	Reutsky-Gefen et al. (2013); Bedbrook et al. (2019); Kosugi et al. (2025); Chiong et al. (2021)
Magnetogenetics	4	2	4	1	±2	1	2	Qin et al. (2016); Del Sol-Fernández et al. (2022); Long et al. (2015); Zhang et al. (2026)
Sonogenetics	4	4	2	1	±2	3	1	Ibsen et al. (2015); Darmani et al. (2025); Xu et al. (2023); Geng et al. (2020); Qiu et al. (2017)

**TABLE 3:** Clinical Application Scenarios, Core Requirements, and Scoring Logic for the Five Genetic Manipulation Technologies Based on the established scoring criteria, quantitative scoring was performed for the five genetic manipulation technologies, wherein a score of five indicates optimal adaptation, three denotes basic adaptation, two represents partial adaptation with limitations, and 1 signifies poor adaptation thereby furnishing researchers with a direct and quantitative reference for rapidly screening optimal technical solutions tailored to their practical research needs (Figure 3).

Application scenario	Core requirements	Scoring logic (5/3/1 points)
Neural Circuits (Basic Research)	High spatiotemporal resolution; precise modulation (neuron localization, real-time regulation)	5 pts: Millisecond-scale precise modulation (optogenetics) 3 pts: Moderate precision (magnetogenetics/sonogenetics, lower spatiotemporal resolution) 1 pt: Low resolution (odorgenetics/chemogenetics, molecular diffusion time lag)
Deep Brain Region Modulation	Strong tissue penetration (skull/deep brain access, reduced invasiveness)	5 pts: Extreme penetration (magnetogenetics/sonogenetics, non-invasive deep reach via magnetic fields/ultrasound) 3 pts: Moderate penetration (chemogenetics, ligand blood-brain barrier diffusion) 1 pt: Poor penetration (optogenetics/odorgenetics, limited light/odor coverage)
Local Tumor Therapy	Penetration + targeting + low invasiveness (tumor access, precise modulation, minimal normal tissue damage)	5 pts: Strong penetration + excellent targeting (sonogenetics, ultrasound-targeted tumor activation, low damage) 3 pts: Moderate penetration (magnetogenetics: strong penetration/weak targeting; chemogenetics: local ligand delivery) 1 pt: Poor penetration/targeting (optogenetics: poor light penetration; odorgenetics: weak odorant targeting)
Respiratory Diseases (Asthma)	Delivery convenience + organ specificity (airway signal delivery, adaptation to respiratory physiology)	5 pts: Extreme delivery convenience (odorgenetics, direct inhaled odorants acting on airways) 3 pts: Moderate convenience (chemogenetics, nebulized administration, weak specificity) 1 pt: Inconvenient delivery (optogenetics/magnetogenetics/sonogenetics, equipment-dependent, no direct respiratory action)
Chronic Metabolic Diseases	Sustained modulation + biosafety (long-term mild regulation, reduced frequent intervention)	5 pts: Strong sustained modulation (chemogenetics, long-term ligand administration, prolonged efficacy) 3 pts: Moderate sustainability (odorgenetics/magnetogenetics/sonogenetics, regular intervention, minimal trauma) 1 pt: Poor sustainability (optogenetics, continuous illumination, high long-term trauma)
Clinical Emergency Intervention	Rapid onset (fast modulation initiation/termination, short response time)	5 pts: Instant onset (optogenetics, millisecond-scale optical signal on/off, rapid abnormal activity termination) 3 pts: Moderate onset speed (odorgenetics/magnetogenetics/sonogenetics, signal/molecular diffusion time) 1 pt: Delayed onset (chemogenetics, ligand metabolism/absorption, 10+ mins to hours for efficacy)
Large-Scale Animal Experiments	Operational simplicity + low invasiveness (batch animal processing, reduced operational complexity)	5 pts: Extreme operational simplicity (sonogenetics, batch ultrasound modulation, no implantation) 3 pts: Moderate simplicity (magnetogenetics/odorgenetics/chemogenetics, no complex implantation/convenient administration) 1 pt: Cumbersome operation (optogenetics, fiber implantation per animal, difficult large-scale processing)

**FIGURE 3:**
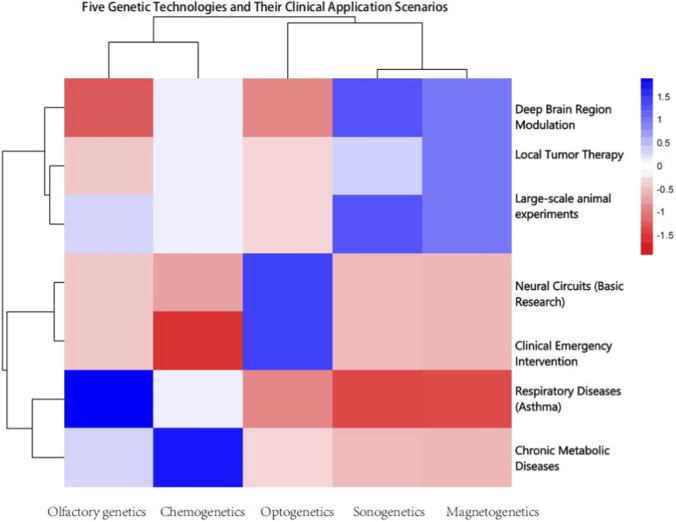
Heatmap of association between five genetic manipulation technologies and their clinical application scenarios.

This heatmap visualizes the quantitative adaptation associations between five genetic manipulation technologies (columns: Olfactory genetics, Chemogenetics, Optogenetics, Sonogenetics, Magnetogenetics) and seven clinical application scenarios (rows: as labeled on the right). The color gradient (ranging from −1.5 (deep red) to 1.5 (deep blue)) corresponds to the strength of scenariotechnology adaptation: deeper blue indicates higher adaptation suitability, while deeper red denotes lower adaptation compatibility, consistent with the scoring criteria outlined in Section 3.1. The hierarchical clustering (dendrograms) on the axes represents a distance metric, grouping technologies and application scenarios based on the similarity of their adaptation profiles.

### 4.2 Detailed analysis of core application scenarios for each technology

#### 4.2.1 Optogenetics

Leveraging the precise manipulation mechanism of lightsensitive proteins with specific wavelengths of light, optogenetics dominates application scenarios demanding high spatiotemporal resolution—particularly in basic neural circuit research and clinical emergency intervention36. In cuttingedge neuroscience research, optogenetics has been established as the exclusive tool for investigating rapid neural processes (e.g., neural coding, circuit synchrony): researchers can activate or inhibit specific neurons with millisecond-scale precision, monitor neural signal transmission pathways in real time, and furnish critical data for dissecting the brain’s complex functional networks (Kim et al., 2017).

In clinical applications, optogenetics exhibits substantial potential for epilepsy treatment: by delivering light-sensitive proteins into hyperexcitable neurons, abnormal electrical discharges can be suppressed via specific-wavelength illumination prior to seizure onset, enabling effective control of epileptic seizures (Krook-Magnuson et al., 2013). Additionally, in neuromodulation research for movement disorders (e.g., Parkinson’s disease), optogenetics also demonstrates significant value in the precise regulation of neural signal conduction.

#### 4.2.2 Chemogenetics

Relying on the regulatory system of Designer Receptors Exclusively Activated by Designer Drugs (DREADDs), chemogenetics possesses irreplaceable advantages in application scenarios requiring long-term, stable modulation—especially in the management of chronic metabolic diseases. The DREADDs system provides temporal control ranging from minutes to hours, making it particularly suitable for investigating long-term homeostatic regulatory processes (e.g., learning, emotion, sleep) (Kurogi et al., 2024).

In metabolic disease research, researchers have successfully elucidated key signaling pathways underlying the maintenance of normal glucose and energy homeostasis by activating or inhibiting metabolism-related neurons via chemogenetic techniques (Ruud et al., 2017). For instance, in animal models of Type 2 diabetes mellitus, modulating hypothalamic appetiteregulating neurons using chemogenetics not only revealed the intrinsic link between glycometabolism and neural regulation, but also provided a theoretical basis for the development of novel therapeutic strategies for metabolic diseases. Meanwhile, in research on the pathological mechanisms of obesity, chemogenetics has been employed to explore the crosstalk between lipid metabolism and the central nervous system (Moullé et al., 2014).

#### 4.2.3 Magnetogenetics and sonogenetics

Magnetogenetics and sonogenetics share a common core advantage of strong tissue penetration capability, rendering them particularly suitable for deep brain region modulation (Chen et al., 2021b). Magnetogenetics utilizes the interaction between magnetic nanoparticles and magnetic fields, featuring distinctive merits including non-invasiveness, high penetration depth, prolonged stimulation duration, spatial unrestriction, spatial uniformity, and relative biosafety. Unlike traditional electrical stimulation that requires electrode implantation, this technology enables remote modulation of deep brain region neurons via external magnetic fields, exhibiting great potential for the treatment of refractory diseases such as depression and addiction disorders.

Sonogenetics, characterized by its non-invasiveness, high precision, and deep tissue penetration, serves as an ideal option for deep brain region modulation. By virtue of ultrasound technology, sonogenetics can convert acoustic energy into intracellular mechanical signals to precisely activate neurons in specific regions. In research on deep brain stimulation therapy for Parkinson’s disease, sonogenetics has achieved non-invasive modulation of neuronal activity in the medial globus pallidus, offering a new direction for the development of safer and more effective therapeutic approaches. Beyond neurological disorders like Parkinson’s disease, the clinical application scenarios for sonogenetics have recently expanded into the cutting-edge field of tumor immunotherapy. Latest research has demonstrated the powerful combination of sonogenetics with engineered immune cells (e.g., CAR-T cells) or therapeutic bacteria. By utilizing focused ultrasounds to non-invasively penetrate solid tumors, researchers can precisely activate mechanosensitive channels engineered into immune cells residing within the tumor microenvironment (Kennedy, 2005). This targeted spatiotemporal activation triggers the localized release of cytokines or therapeutic proteins, effectively overcoming the immunosuppressive tumor microenvironment while minimizing systemic off-target toxicity (Forbes, 2010; Nissim et al., 2017). This breakthrough significantly broadens the translational scope of sonogenetics from pure neuromodulation to precision oncology.

#### 4.2.4 Odorgenetics

Based on the regulatory system of Odor-activated Designer Receptors (ODRs), odorgenetics exhibits unique advantages in modulation scenarios requiring precision, non-invasiveness, and spatiotemporal controllability—particularly in the fields of neural circuit function dissection, behavioral regulation, and related disease intervention. The ODRs system employs specific odorant molecules as activation signals, achieving a regulatory window ranging from second-scale responses to sustained modulation lasting several hours. It not only avoids potential systemic side effects associated with chemical drugs but also overcomes the invasive limitations of optogenetics that rely on optical fiber implantation, making it especially suitable for investigating complex physiological processes (e.g., olfactory perception, emotional correlation, memory formation).

In neuropsychiatric disease research, researchers have successfully revealed the intrinsic regulatory network between olfactory signals and emotional/cognitive functions by specifically activating or inhibiting neural circuits in distinct brain regions via odorogenetic techniques. For example, in addiction animal models, modulating dopaminergic neuronal circuits in the nucleus accumbens using odorgenetics not only clarified the neural mechanisms underlying the alleviation of addiction-like behaviors, but also provided critical experimental evidence for the development of non-invasive intervention strategies for neuropsychiatric diseases. Additionally, in research on the pathological mechanisms of Alzheimer’s disease, odorgenetics has been used to explore the association between olfactory pathway degeneration and memory impairment; targeted activation of olfactory bulbhippocampal neural projections effectively improved memory retrieval ability in model animals, offering novel technical insights for the early intervention of this disease.

### 4.3 Integration with neural activity recording and imaging methods

In modern systems neuroscience, genetic manipulation technologies are rarely used in isolation; rather, they are integrated with advanced neural recording and imaging techniques to link causal perturbations to circuit dynamics and behavioral outcomes. For instance, optogenetics and chemogenetics are frequently combined with electrophysiology, fiber photometry, or calcium imaging (e.g., using the COMBO window for multiscale circuit interrogation in mice) to monitor real-time cellular responses (Edelman et al., 2024). Additionally, combining these tools with macroscopic hemodynamic imaging has yielded profound insights into brain-wide networks. Recent studies have successfully utilized chemogenetic manipulation alongside resting-state fMRI to reveal the neural basis of functional connectivity in fronto-limbic circuits (Elorette et al., 2024). Similarly, functional ultrasound (fUS) brain imaging provides a powerful, high-resolution modality to bridge networks and behavior during manipulation (Ede et al., 2021). Emerging tools are also being integrated into these paradigms; for example, sonogenetics has been successfully coupled with electrophysiological recordings to validate the precise modulation of deep neural circuits. These combined manipulation-recording paradigms are essential for decoding the complex causality of neural systems.

### 4.4 Technology selection guidance table

Ultimately, the core parameters of the five genetic manipulation technologies were summarized in Table 3. Based on their technical performance, this study presents a guidance table for application scenario selection (Table 4), as well as the current limitations of each technique and directions for further optimization (Table 6).

**TABLE 4:** Summarizes the key technical parameters of the five genetic manipulation technologies, encompassing core components, stimulation modes, spatiotemporal resolution, duration of action, typical advantages, and key limitations. The quantitative and qualitative comparisons presented herein intuitively delineate the distinctive performance characteristics and inherent trade-offs of each technology, thereby facilitating rational technology selection for basic research (e.g., neural circuit dissection, metabolic disease modeling) and clinical translational studies (e.g., deep brain region modulation, local tumor therapy). Based on their technical performance, this study presents a guidance table for application scenario selection (Table 4) and Comparative Summary of Technical Limitations and Future Optimization Avenues (Table 5).

Technology	Core components	Stimulation mode	Spatiotemporal resolution	Duration	Typical advantages	Key limitations
Odorgenetics	DORS receptor, 2-pentanone	Inhalation administration	Minute/mm scale	5–30 min	Non-invasive, rapid reversibility	Limited ligand types
Optogenetics	Optogenetic protein, optical fiber	Blue/yellow light	Millisecond/μm scale	Instantaneous	Highest spatiotemporal resolution	Need for implantation, shallow penetration
Chemogenetics	DREADDs receptor, CNO	Intraperitoneal injection/oral administration	Hour/mm scale	4–24 h	Long-acting stability, easy operation	Non-specificity from ligand metabolism
Magnetogenetics	MagR protein, magnetic nanoparticles	Alternating magnetic field	Minute/cm scale	Several minutes	Deep penetration, fully non-invasive	Low spatial resolution
Sonogenetics	TRP channel, ultrasound transducer	Low-frequency ultrasound	Second/mm scale	Several seconds	Deep penetration, focusable	Potential damage from high-intensity ultrasound

**TABLE 5:** Provides concise technology selection guidance for diverse research requirements in biomedical and nanobiotechnology f ields, specifying the preferred and alternative genetic manipulation technologies along with the corresponding selection rationales. The recommendations are strictly derived from the core performance parameters of each technology (e.g., spatiotemporal resolution, modulation duration, invasiveness), which are systematically analyzed in the preceding sections. This table enables researchers to rapidly match the most suitable technical strategy to their specific experimental objectives, thereby optimizing the design of basic research and clinical translational studies.

Research requirement	Preferred technology	Alternative technology	Selection rationale
Deciphering millisecond-scale neural signal transmission	Optogenetics	Sonogenetics	Optogenetics achieves 0.1 ms temporal resolution, suitable for capturing action potential transmission
Establishing chronic disease models (e.g., depression)	Chemogenetics	Magnetogenetics	Chemogenetics enables sustained modulation for over 24 h, matching disease progression cycles
Behavioral studies in freely moving animals	Odorgenetics	Sonogenetics	Inhalation administration eliminates device restraint, without interfering with natural animal behaviors
Modulation of deep brain regions (e.g., substantia nigra)	Sonogenetics	Magnetogenetics	Ultrasound can be focused to a 0.5 mm^3^ region, superior to magnetogenetics’ cm-scale resolution
Exploration of non-invasive tumor therapy	Sonogenetics	Magnetogenetics	Ultrasound activates engineered bacteria in tumors to release cytokines, avoiding side effects of surgery/radiotherapy

**TABLE 6:** To facilitate a rapid comparison of the developmental hurdles facing each technology, we have compiled a comprehensive summary of their core limitations, current mitigation strategies, and future optimization avenues. This framework highlights the trajectory of the field towards less invasive, more specific, and clinically safer manipulation tools.

Technology	Core limitations	Current mitigation strategies	Future optimization avenues
Odorgenetics	Potential off-target activation of endogenous olfactory receptors; lack of long-term inhalation toxicity data	Using highly specific, non-endogenous volatile ligands (e.g., 2-pentanone)	Engineering mammalian-derived ORs to reduce immunogenicity; developing highly volatile, FDA-approved inert ligands
Chemogenetics	Slow onset/offset kinetics; reverse metabolism of CNO to clozapine causing off-target effects	Development of novel, highly specific ligands (e.g., DCZ, JHU37160) with minimal reverse metabolism	Designing next-generation DREADDs with faster kinetics; integrating localized drug delivery systems
Optogenetics	Poor tissue penetration of visible light; requires invasive surgical implantation of optical fibers	Utilizing red-shifted opsins (e.g., Chrimson) or upconversion nanoparticles (UCNPs) for deeper penetration	Developing ultra-sensitive opsins excitable by transcranial near-infrared (NIR) light; completely wireless optoelectronic implants
Magnetogenetics	Mechanistic controversies regarding magnetic force sufficiency; potential thermal damage from AMFs	Enhancing iron loading in ferritin; using synthetic superparamagnetic nanoparticles	Elucidating exact biophysical transduction mechanisms; engineering high-magnetic-moment biogenic nanoparticles
Sonogenetics	High acoustic intensity required may cause cavitation tissue damage; skull-induced ultrasound aberration	Co-administration of gas vesicles/microbubbles to amplify mechanical signals at lower intensities	Discovering/engineering ultrasound-hypersensitive mammalian channels; advancing patient-specific transcranial acoustic focusing algorithms

## 5 Conclusions and future perspectives

### 5.1 Summary of key findings

This review systematically compared five genetic manipulation technologies—chemogenetics, optogenetics, odorgenetics, magnetogenetics, and sonogenetics—across molecular mechanisms, quantitative performance metrics, and application scenarios. Three principal conclusions emerge from this analysis: First, no single technology excels across all dimensions. Optogenetics provides unmatched spatiotemporal precision but requires invasive light delivery. Chemogenetics offers clinical practicality but sacrifices temporal resolution. Magnetogenetics and sonogenetics enable non-invasive deep tissue access but face mechanistic uncertainties. Odorgenetics presents a promising middle ground with diverse receptor options and accessible ligands, though further optimization is needed. Second, technology selection should be guided by application-specific requirements rather than absolute performance rankings. For millisecond-scale neural circuit dissection, optogenetics remains the gold standard. For chronic neuromodulation in clinical settings, chemogenetics offers the most practical pathway. For non-invasive deep brain stimulation, sonogenetics and magnetogenetics warrant further development. Third, the seven-dimensional evaluation framework introduced here provides a standardized basis for technology comparison that can be updated as new data emerge and extended to future technologies.

### 5.2 Recommendations for technology selection

Based on our analysis, we propose the following decision guidelines:

Prioritize optogenetics when: millisecond temporal precision is essential; superficial tissue access is acceptable; acute experimental paradigms are employed. Prioritize chemogenetics when: sustained modulation over hours to days is required; systemic or oral administration is preferred; clinical translation is a primary goal. Consider odorgenetics when: diverse receptor-ligand combinations are needed; natural compound ligands are preferred; multiplexed control is desired. In review Consider magnetogenetics/sonogenetics when: non-invasive deep tissue access is essential; hardware infrastructure is available; longer-term technical development timelines are acceptable.

### 5.3 Future research directions

Several priorities emerge for advancing the field: (1) Hybrid technologies: Combining the strengths of multiple approaches can achieve unprecedented multi-timescale control. For example, integrating optogenetics (for millisecond-scale, precise initiation of a behavior) with chemogenetics (for sustained, hour-scale maintenance of a physiological state) within the same neural circuit can overcome the temporal limitations of individual tools. Additionally, coupling these manipulation actuators with real-time neural recordings (e.g., electrophysiology or calcium imaging) will enable the development of adaptive, closed-loop neural control systems, where cellular manipulation is automatically triggered by specific pathological biomarkers (e.g., seizure onset). (2) Enhanced actuators: Development of red-shifted opsins for deeper penetration, high-affinity magnetoreceptors, and engineered odorant receptors with improved specificity. (3) Delivery innovations: non-viral vectors, cell-specific promoters, and inducible expression systems to improve safety and specificity. (4) Computational integration: Machine learning approaches for predicting optimal stimulation parameters and receptor-ligand combinations. (5) Standardized benchmarking: Establishment of community wide standards for reporting performance metrics to enable rigorous cross-study comparisons. 5.4 Concluding remarks Genetic manipulation technologies have transformed neuroscience and are poised to impact clinical medicine. The comparative framework presented here aims to guide researchers in navigating this expanding toolkit. As these technologies mature, their integration with advanced imaging, computation, and delivery platforms will unlock new possibilities for understanding and treating neurological and psychiatric disorders.
